# Blood Pressure Levels in Male Carriers of Arg82Cys in *CD300LG*

**DOI:** 10.1371/journal.pone.0109646

**Published:** 2014-10-14

**Authors:** Julie Støy, Niels Grarup, Arne Hørlyck, Liselotte Ibsen, Jørgen Rungby, Per Løgstrup Poulsen, Ivan Brandslund, Cramer Christensen, Torben Hansen, Oluf Pedersen, Niels Møller, Ulla Kampmann

**Affiliations:** 1 Department of Internal Medicine and Endocrinology, Aarhus University Hospital, Aarhus, Denmark; 2 The Novo Nordisk Foundation Center for Basic Metabolic Research, Faculty of Health and Medical Sciences, University of Copenhagen, Copenhagen, Denmark; 3 Department of Radiology, Aarhus University Hospital, Aarhus, Denmark; 4 Department of Endocrinology, Rigshospitalet, Copenhagen, Denmark; 5 Department of Clinical Biochemistry, Vejle Hospital, Vejle, Denmark; 6 Department of Medicine, Vejle Hospital, Vejle, Denmark; 7 Medical Research Laboratories, Institute for Clinical Medicine, Aarhus University, Aarhus, Denmark; University of Utah School of Medicine, United States of America

## Abstract

The genetics of hypertension has been scrutinized in large-scale genome-wide association studies (GWAS) with a large number of common genetic variants identified, each exerting subtle effects on disease susceptibility. An amino acid polymorphism, p.Arg82Cys, in *CD300LG* was recently found to be associated with fasting HDL-cholesterol and triglyceride levels. The polymorphism has not been detected in hypertension GWAS potentially due to its low frequency, but *CD300LG* has been linked to blood pressure as *CD300LG* knockout mice have changes in blood pressure. Twenty-four-hour ambulatory blood pressure was obtained in human *CD300LG* CT-carriers to follow up on these observations.

**Methods:**

Twenty healthy male *CD300LG* rs72836561 CT-carriers matched for age and BMI with 20 healthy male CC-carriers. Office blood pressure, 24-hour ambulatory blood pressure, carotid intima-media thickness (CIMT), and fasting blood samples were evaluated. The clinical study was combined with a genetic-epidemiological study to replicate the association between blood pressure and *CD300LG* Arg82Cys in 2,637 men and 3,249 women.

**Results:**

CT-carriers had a higher 24-hour ambulatory systolic blood pressure (122 mmHg versus 115; p = 0.01) and diastolic blood pressure (77 mmHg versus 72; p<0.01) compared with CC-carriers. There were no differences in CIMT between the two groups. Metalloproteinase-9 level was higher in CT-carriers than in CC-carriers (P<0.01). However, no association between office blood pressure and *CD300LG* genotype was detected in the genetic-epidemiological study.

**Conclusions:**

Although 24-hour blood pressure, measured with a sensitive method, in a small sample of *CD300LG* rs72836561 CT-carriers was higher than in CC-carriers, this did not translate into significant differences in office blood pressure in a larger cohort. This discrepancy which may reflect differences in methodological approach, underlines the importance of performing replication studies in a larger clinical context, but a formal rejection of a relation between blood pressure and *CD300LG* requires measurement of 24-hour ambulatory blood pressure in a larger cohort.

## Introduction

Hypertension constitutes an important public health challenge due to its association with cardiovascular diseases [1], [2]. Hypertension often coexists with metabolic disorders such as obesity, dyslipidaemia, and glucose intolerance. Each of these disorders has been scrutinized in genetic association studies with a large number of genetic variants identified that exert a subtle effect on disease susceptibility [3]–[6]. The possibility that the metabolic disorders on top of their phenotype-specific genes may share overlapping causative genes was recently explored in a 3 stage genetic–epidemiological study combining whole exome sequencing of 2000 Danish individuals (stage 1) with genotyping and association studies in Danish (stage 2) and European (stage 3) individuals [7]. An amino acid polymorphism in *CD300LG* (c.313 C>T, p.Arg82Cys) with a minor allele frequency of 3.5% was selected for replication in stage 2 and 3 of the study due to its annotation as a missense mutation in stage 1 of the study. In stage 2 and 3 of the study, the polymorphism was associated with a decreased fasting HDL-cholesterol level and an increased fasting triglyceride level. The protein encoded by *CD300LG* is a membrane-bound protein expressed on vascular endothelial cells in a broad range of human tissues with the highest expression in placenta, adipose tissue and skeletal muscle [8]–[13]. The biological functions of *CD300LG* are not well characterized, but *CD300LG* knockout (KO) mice have osteopenia and decreased systolic blood pressure (US patent number 2008/0311107A1, Bollinger et al.). In the present study, aspects of the cardiovascular phenotype were characterized in healthy male heterozygous carriers of the *CD300LG* rs72836561 polymorphism to address the hypothesis that *CD300LG* has effects on blood pressure regulation as suggested in studies of the *CD300LG* KO mice.

## Materials and Methods

### Study participants

Twenty healthy male *CD300LG* rs72836561 CT-carriers and 20 age and BMI matched CC-carriers (controls) were recruited from Biobank Vejle [7]. The 40 study subjects were examined over two days with the aim of characterizing the metabolic profile of carriers of the *CD300LG* T-allele, but in the present report only data on the cardio-vascular phenotype were included. Only males were included in the study to avoid the effect of the menstrual cycle on the metabolic variables. The study subjects gave a written informed consent prior to study participation. The study protocol was approved by the regional ethical committee (Region Midt, 1-10-72-113-12) and the studies were conducted according to the principles of the Helsinki declaration.

### Design

After an overnight fast, body composition was evaluated by DXA-scan (Hologic QDR 2000). An intravenous catheter was inserted into a cubital vein for sampling of peripheral blood. Blood samples were drawn after 90 minutes of bed rest. 24-hour blood pressure and heart rate were obtained using an oscillometric blood pressure monitor (Spacelab 91270, Washington, USA). Blood pressure and heart rate were recorded every 20 minutes for 24 hours and classified into daytime or nighttime recordings according to information from the study subjects. Carotid intima-media thickness (CIMT) was obtained by ultrasonography. Two radiologists used the same protocol to obtain bilateral B-mode images during the cardiac diastole of the far wall of the common carotid artery and in the bulb of the internal carotid artery using dedicated software (H4910MT, L E9 Automatic IMT Measurement Option). The radiologists were blinded to the genotype of the study subjects.

### Assays

Pro-Brain natriuretic peptide (pro-BNP) was analyzed using an immunometric method. V-CAM1, I-CAM1, metalloproteinase-9 (MMP-9), high-sensitive C-reactive protein (HsCRP), aldosterone, and renin were analyzed by ELISA using commercially available kits (Bio-rad, Berkeley, California, USA and R&D Systems Europe Ltd., Abingdon, UK). Plasma free metanephrine and normetanephrine were analyzed using a commercially available enzyme immunoassay (EIA, Labor Diagnostika Nord Gmbh and CO. KG, Nordhorn, Germany).

### Replication analyses of genetic-epidemiological data

With the aim of replicating the results from the clinical study, a post-hoc analysis of the blood pressure data from the original Danish genetic-epidemiological study of 15,989 Danish individuals was performed [7]. Subjects with a phenotype resembling the participants in the clinical study were included: non-obese, non-diabetic men in the age interval 37-69 years. Fulfilling these criteria office blood pressure was available for a total of 2,475 *CD300LG* CC-carriers, 157 CT-carriers, and 5 TT-carriers. As blood pressure changes had been observed in female *CD300LG* KO mice, we also tested the association between *CD300LG* Arg82Cys and office blood pressure in women using data from the original genetic-epidemiological study. Data were available for 3,028 female *CD300LG* CC-carriers, 218 CT-carriers, and 3 TT-carriers.

### Statistical analysis

Statistical analyses were performed using SPSS 21.0 and R 2.12. Results are presented as mean +/- 95% confidence interval or median +/− interquartile range. A Students t-test was applied to compare the two genotype groups. The Kolmogorov-Smirnov test was used to test whether data were normally distributed. P-values <0.05 was considered significant. A multiple regression model was applied to adjust blood pressure measurements for age and fasting triglyceride level (TAG). The analyses of CIMT included adjustment for age and 24-hour systolic blood pressure. Bivariate associations of continuous variables were tested using Pearson's coefficient of correlation. Variables were omitted from the regression model if P>0.1. A Fisher's exact test was used to test for association between dichotomous variables. In a post hoc power calculation with the 24-hour systolic blood pressure as endpoint, the statistical power was 80% to detect a difference of 7,5 mmHg with 20 subjects in each study group. In the genetic-epidemiological replication study a linear regression analysis was performed applying an additive genetic model adjusted for age. Outcome variables were rank normalized before analysis. The choice of an additive genetic model was arbitrary as we had no prior knowledge of the effect of the minor allele on risk transmission.

## Results

### Anthropometrics

Clinical characteristics of the study subjects are presented in table 1. There were 5 current smokers in the CT-group compared to one current smoker in the CC-group. Three subjects were treated with antihypertensive drugs; doses were omitted at least 72 hours prior to the study day. The analyses of blood pressure and CIMT were repeated after exclusion of current smokers and subjects receiving anti-hypertensive drugs and this did not change the results (tables S1 and S2).

**Table 1:** Data on the study subjects according to *CD300LG* rs72836561 CC, CT, and TT genotype.

Clinical study	CC (n = 20)	CT (n = 19)	TT	P
**Anthropometric data**				
Age (years)	55.1 (50.8–59.2)	55.0 (50.8–59.3)	-	0.99
BMI (kg/m^2^)	24.6 (23.8–25.5)	24.5 (23.4–25.6)	-	0.79
Lean body mass (kg)	59.0 (56.7–61.3)	58.3 (55.3–61.4)	-	0.72
Fat mass (kg)	16.9 (15.1–18.6)	16.9 (15.0–18.8)	-	0.99
Office systolic blood pressure (mmHg)	130 (126–133)	140 (132–147)	-	0.02
Office diastolic blood pressure (mmHg)	78 (75–82)	84 (81–87)	-	0.02
**Fasting biochemical profile**				
HbA1c (IFCC, mmol/mol)	35.2 (33.8–36.5)	35.7 (34.5–36.9)	-	0.56
Glucose (mmol/l)	5.5 (5.3–5.6)	5.5 (5.3–5.7)	-	0.68
Total cholesterol (mmol/l)	5.3 (5.0–5.6)	5.1 (4.7–5.4)	-	0.18
Low density lipoprotein (LDL, mmol/l)	3.4 (3.1–3.7)	3.1 (2.8–3.4)	-	0.19
High density lipoprotein (HDL, mmol/l)^1^	1.4 (1.2–1.7)	1.2 (1.0–1.5)	-	0.28
Triglyceride (mmol/l) 1	1.1 (0.8–1.4)	1.3 (0.8–1.6)	-	0.34
**24-hour ambulatory blood pressure**				
*Systolic blood pressure (mmHg)*				
24-hour	115 (111–118)	122 (117–127)	-	0.01
Daytime	120 (117–124)	128 (123–133)	-	0.01
Nighttime	102 (98–106)	108 (101–114)	-	0.10
*Diastolic blood pressure (mmHg)*				
24-hour	72 (70–74)	77 (74–80)	-	0.01
Daytime	76 (74–78)	81 (79–84)	-	<0.01
Nighttime	62 (59–64)	65 (61–69)	-	0.08
**Biochemical profile**				
Aldosterone/renin ratio^1^	277 (218–489)	521 (306–743)	-	0.10
Metaneprine (pg/ml)^1^	33 (28–40)	33 (23–42)	-	0.89
Normetanephrine (pg/ml)^1^	27 (22–31)	25 (20–30)	-	0.91
ICAM-1 (ng/ml)	56.8 (32.8–148.1)	58.3 (31.3–91.0)	-	0.82
VCAM-1 (ng/ml)	78.6 (38.3–172.4)	77.4 (32.3–111.5)	-	0.24
MMP9 (ng/ml)^1^	560 (401–704)	685 (565–1009)	-	0.01
Pro-BNP (pg/ml)^1^	27.0 (9.2–51.7)	22.7 (7.2–64.8)	-	0.70
HsCRP (mg/l)^1^	1.35 (0.39–3.63)	1.24 (0.72–2.64)	-	0.80
**Genetic-epidemiological study**				
**Anthropometric data (men)**	CC (n = 2,475)	CT (n = 157)	TT (n = 5)	
Age (years)^2^	49.9 (44.8–55.0)	50.0 (44.9–55.0)	44.9 (44.8–45.0)	-
BMI (kg/m^2^) 2	25.4 (23.5–27.3)	25.5 (23.8–27.5)	23.4 (23.1–24.9)	0.52
Office systolic blood pressure (mmHg) 2	130 (120–140)	130 (120–140)	145 (130–152)	0.78
Office diastolic blood pressure (mmHg) 2	84 (79–90)	83 (78–90)	90 (70–100)	0.59
**Fasting biochemical profile**				
HbA1c (%)^2^	5.7 (5.4–6.0)	5.7 (5.5–6.0)	5.9 (5.5–5.9)	0.38
Glucose (mmol/l) 2	5.5 (5.2–5.9)	5.7 (5.3–5.9)	5.6 (5.4–5.6)	0.15
Total cholesterol (mmol/l) 2	5.5 (4.9–6.2)	5.6 (4.9–6.2)	5.6 (5.5–6.3)	0.25
High density lipoprotein (HDL, mmol/l) 2	1.4 (1.1–1.6)	1.3 (1.1–1.6)	1.3 (1.3–1.4)	0.43
Triglyceride (mmol/l) 2	1.1 (0.8–1.6)	1.1 (0.9–1.6)	1.2 (1.1–2.0)	0.40
**Anthropometric data (women)**	CC (n = 3,028)	CT (n = 218)	TT (n = 3)	
Age (years)^2^	49.9 (44.1–55.0)	49.7 (44.0–55.0)	53.0 (47.0–54.1)	-
BMI (kg/m^2^) 2	23.8 (21.8–26.0)	23.6 (21.9–25.7)	20.8 (20.3–23.0)	0.76
Office systolic blood pressure (mmHg) 2	123 (114–135)	120 (114–135)	119 (110–127)	1.00
Office diastolic blood pressure (mmHg) 2	80 (72–85)	80 (70–85)	70 (66–73)	0.95
**Fasting biochemical profile**				
HbA1c (%)^2^	5.6 (5.3–5.9)	5.6 (5.3–5.8)	5.3 (5.2–5.7)	0.74
Glucose (mmol/l) 2	5.3 (5.0–5.6)	5.2 (5.0–5.6)	5.0 (4.7–5.4)	0.78
Total cholesterol (mmol/l) 2	5.4 (4.8–6.1)	5.2 (4.6–6.0)	5.3 (5.2–5.4)	0.02
High density lipoprotein (HDL, mmol/l) 2	1.7 (1.4–1.9)	1.6 (1.4–1.8)	1.7 (1.6–1.7)	0.03
Triglyceride (mmol/l) 2	0.9 (0.7–1.2)	1.0 (0.7–1.3)	1.2 (0.9–1.3)	0.16

Mean and 95% confidence interval. P-values obtained from Students t-test.

1 Median and interquartile range. P-value obtained from Students t-test of log-transformed data.

2 Median and interquartile range. P-value obtained from linear regression on rank normalized data adjusted for age.

### Blood pressure measurements

Study subjects from the CT-group had significantly higher 24-hour and daytime ambulatory blood pressure compared to the controls in the CC-group. Nighttime measurements displayed the same trend with higher blood pressure in the CT-group, but the differences were not statistically significant. Adjusting blood pressure for age and fasting triglyceride level did not change the results. There were no differences between the two groups in heart rate, circadian variation in heart rate, pulse pressure or nighttime dipping (results not shown). One subject from the CT-group was excluded from the analyses due to manifest hypertension (24-hour average 161/100) to avoid skewing of the data.

### Carotid intima-media thickness

There were no differences in CIMT measures between the CT-group and the CC-group (table S2). Adjusting the data for age and 24-hour systolic blood pressure did not change the results.

### Biochemical analyses

To explore the physiological mechanisms underlying the higher blood pressure levels in the CT-group, a biochemical profile relevant to the cardiovascular phenotype was obtained. The panel of vascular endothelial markers revealed significantly higher levels of MMP9 in the CT-group than in the CC-group (P<0.01). There was a tendency to higher aldosterone/renin ratio in the CT-group than in the CC-group, but this difference was not statistically significant (P = 0.10).

### Replication analysis of genetic epidemiological data

The findings of the clinical study were not confirmed in the replication study of office blood pressure versus *CD300LG* genotype; in the linear regression, the beta-coefficient for systolic blood pressure was 0.003 (p = 0.75) and for diastolic blood pressure −0.005 (p = 0.56) in men and the beta-coefficient for systolic blood pressure was −3.17*10^−5^(p = 1.00) and 0.004 for diastolic blood pressure in women (p = 0.95), respectively.

## Discussion

In the present study, healthy male *CD300LG* rs72836561 CT-carriers had higher levels of 24-hour and daytime systolic and diastolic ambulatory blood pressures than age and BMI matched CC-carriers. *CD300LG* rs72836561 was originally identified in a genetic-epidemiological study [7] due to its association with decreased fasting HDL-cholesterol levels and increased fasting triglyceride levels whereas no association between *CD300LG* and office blood pressure was detected. The cohorts used for the genetic-epidemiological study included subjects with obesity and diabetes and both sexes were included in the analyses. After finding the effect of *CD300LG* on 24-hour blood pressure in the clinical study, the data from the original genetic-epidemiological study was filtered with the aim of re-analyzing data with inclusion of only men with a phenotype resembling the men in the clinical study. In this analysis we found no association between *CD300LG* and systolic office blood pressure (p = 0.75) nor diastolic office blood pressure (p = 0.56). Unfortunately, 24-hour ambulatory blood pressure measurements were unavailable in the genetic-epidemiological data, but the high number of study subjects potentially compensated for the less precise methodology. In the clinical study, systolic office blood pressure correlated well with 24-hour ambulatory systolic blood pressure (r = 0.65, p<0.01) whereas the correlation was weaker for ambulatory diastolic blood pressure (r = 0.37, p = 0.02), but in larger studies both systolic and diastolic office blood pressure have been shown to correlate well with ambulatory blood pressure (r = 0.66) [14]. Earlier studies have shown that 24-hour ambulatory blood pressure is superior to office blood pressure to detect significant differences in blood pressure levels related to genetic variance [15]. We were unable to include subjects that were homozygous for the *CD300LG* minor allele in the clinical study and in the genetic-epidemiological study only a total of 8 homozygotes were included. Consequently, with the present data we were unable to reliably assess the effect of homozygosity for the *CD300LG* minor allele on blood pressure level. In the present study only the potential association between this specific variant in *CD300LG* and blood pressure level was tested. The results may have been influenced by other rare variants in the gene or variants in genes not tested in the present study. Consequently, a formal rejection of a relation between blood pressure and *CD300LG* would require measurement of 24-hour ambulatory blood pressure in a large study cohort.

The participants in the genetic-epidemiological study were slightly younger than the participants in the clinical study. If the potential effect of the *CD300LG* polymorphism on blood pressure becomes manifest with increasing age, then this age difference could potentially mask an effect on blood pressure in the genetic-epidemiological study. Consequently, we performed a sub-analysis in the genetic-epidemiological cohort with inclusion of participants with a similar median age (n = 1,868). This did however not change the results. Likewise, we observed no association between age and blood pressure in the clinical study. The observed differences in blood pressure between the genotype groups were statistically significant. The large differences in blood pressure could, however, be a consequence of recruitment bias with study subjects in the CC-group with 24-hour blood pressure in the lower range of normal blood pressure and/or study subjects in the CT-group with 24-hour ambulatory blood pressure in the higher range of normal 24-hour ambulatory blood pressure. Furthermore, incidental recruitment of an unhealthy CT-group could potentially lead to similar results. With respect to body composition, fasting glucose, and fasting lipid profile we observed no differences between the groups and as such the CT-group seemed at least as healthy as the controls in the CC-group. We assessed the level of physical activity of the study subjects at work and in the leisure time by use of a questionnaire and we observed no clear differences between the study groups.

In the murine *CD300LG* KO model, female mice had decreased systolic blood pressures, but presumably no changes in blood pressure were observed in male mice. We included an analysis of office blood pressure in 3,249 women and tested its association to the *CD300LG* polymorphism. As in men, again we found no association between office blood pressure and *CD300LG* genotype. The effects on protein function of the amino acid change in *CD300LG* has not been characterized, and the observed discrepancy between human carriers of the *CD300LG* polymorphism and the *CD300LG* KO mice may relate to different expression patterns of *CD300LG* and/or a functional difference between having a *CD300LG* with an altered amino acid sequence and having no *CD300LG* protein. We sought to explore the underlying physiology of the observed blood pressure differences by obtaining a biochemical profile. The level of MMP9 was higher in CT-carriers than in CC-carriers and we observed a trend towards a higher aldosterone to renin ratio among CT-carriers whereas the remaining biochemical markers were at the same level in the two groups. MMP9 is involved in remodeling of the extracellular matrix (ECM), physiological processes that act to maintain structural stability of the ECM, due to its ability to degrade collagen[16]. Whether altered MMP9 function is secondary to the vascular remodeling of the resistance arteries as seen in hypertension or MMP9 contributes to the development of hypertension by inducing vascular remodeling is incompletely understood. The observed changes in MMP9 in the present study may thus be secondary to the changes in blood pressure, but a direct effect on blood pressure level is also possible. Due to its effects on sodium reabsorption in the distale tubules of the kidney, aldosterone is a central hormone in the regulation of blood pressure. A slight increase in aldosterone activity may contribute to the observed blood pressure changes in CT-carriers. Although in absolute numbers, the effect of the *CD300LG* variant on fasting HDL-cholesterol and triglyceride was subtle in the genetic-epidemiological gene-discovery study, the effect of the gene variant on fasting HDL-cholesterol was higher than all but one of the previously GWAS-identified HDL-cholesterol-associated variants [3]. We were unable to detect significant differences in HDL-cholesterol and triglyceride in the male study subjects in the present study, most likely due to lack of statistical power.

Carotid intima-media thickness is widely used in clinical and epidemiological research for detection of early cardiovascular disease [17]. Given the substantial differences in blood pressure between the two study groups, we would expect to find differences in CIMT. However, since both groups had fairly low 24-hour blood pressure levels, a significant increase in CIMT might not be detectable. In addition it could be due to lack of statistical power that we were unable to find a statistical significant difference between the two study groups.

## Conclusions

In this study of blood pressure level in carriers of the Arg82Cys polymorphism in *CD300LG,* we found a higher level of 24-hour and daytime systolic and diastolic ambulatory blood pressure in CT-carriers compared with CC-carriers. We combined the clinical study with a genetic-epidemiological study based on a post-hoc analysis of office blood pressure in 2,637 men and 3,249 women. In this large group of subjects, the association could not be confirmed. The findings of the present study underscore the importance of replicating findings from clinical studies in larger and external study cohorts, but a formal rejection of an association between *CD300LG* and blood pressure would require use of a precise methodology such as 24-hour ambulatory blood pressure in a large cohort.

## Supporting Information

Table S1 **Data on the study subjects according to** ***CD300LG*** **rs72836561 CC and CT genotype.**
(DOCX)

Table S2 **Carotid intima-media thickness (CIMT) in the common carotid artery (CCA) and internal carotid artery (ICA) according to** ***CD300LG*** **rs72836561 CC and CT genotype (in mm).**
(DOCX)
