# Supplementary material for: Blood Pressure Levels in Male Carriers of Arg82Cys in CD300LG
Source: PLoS One. 2014 Oct 14;9(10):e109646. doi: 10.1371/journal.pone.0109646 (PMC4196928; doi:10.1371/journal.pone.0109646)
Supplement: Table S1 — Data on the study subjects according to CD300LG rs72836561 CC and CT genotype. (DOCX) [file pone.0109646.s001.docx]

Table S1. Data on the study subjects according to *CD300LG* rs72836561 CC and CT genotype

|  | CC (n=19) | CT (n=15) | P-value |
| --- | --- | --- | --- |
| **24-hour ambulatory blood pressure** |  |  |  |
| After exclusion of current tobacco smokers |  |  |  |
| *Systolic blood pressure (mmHg)* |  |  |  |
| 24-hour | 114.5 (110.9-118.2) | 123.5 (117.4-129.5) | <0.01 |
| Daytime | 119.9 (116.1-123.8) | 130.0 (124.1-135.9) | <0.01 |
| Nighttime | 101.8 (97.7-106.0) | 107.6 (100.0-115.2) | 0.15 |
| *Diastolic blood pressure (mmHg)* |  |  |  |
| 24-hour | 71.6 (69.8-73.5) | 76.9 (73.2-80.7) | <0.01 |
| Daytime | 75.9 (74.0-77.8) | 81.9 (78.3-85.4) | <0.01 |
| Nighttime | 61.4 (59.1-63.8) | 64.6 (59.8-69.4) | 0.18 |
| **24-hour ambulatory blood pressure** | CC (n=19) | CT (n=17) |  |
| After exclusion of subjects treated with an anti-hypertensive drug |  |  |  |
| *Systolic blood pressure (mmHg)* |  |  |  |
| 24-hour | 114.2 (111.0-117.7) | 122.5 (117.0-128.0) | 0.01 |
| Daytime | 120.1 (116.2-124.0) | 128.9 (123.4-134.3) | <0.01 |
| Nighttime | 100.8 (97.8-104.4) | 107.1 (100.2-113.9) | 0.09 |
| *Diastolic blood pressure (mmHg)* |  |  |  |
| 24-hour | 71.8 (69.9-73.7) | 76.6 (73.2-79.9) | 0.01 |
| Daytime | 76.4 (74.4-78.4) | 81.5 (78.4-84.7) | <0.01 |
| Nighttime | 61.1 (58.9-63.2) | 64.5 (60.2-68.9) | 0.13 |

Mean and 95 % confidence interval. P-values obtained from Students t-test.
