# Supplementary material for: Blood Pressure Levels in Male Carriers of Arg82Cys in CD300LG
Source: PLoS One. 2014 Oct 14;9(10):e109646. doi: 10.1371/journal.pone.0109646 (PMC4196928; doi:10.1371/journal.pone.0109646)
Supplement: Table S2 — Carotid intima-media thickness (CIMT) in the common carotid artery (CCA) and internal carotid artery (ICA) according to CD300LG rs72836561 CC and CT genotype (in mm). (DOCX) [file pone.0109646.s002.docx]

Table S2. Carotid intima-media thickness (CIMT) in the common carotid artery (CCA) and internal carotid artery (ICA) according to *CD300LG* rs72836561 CC and CT genotype (in mm).

|  | CC (n=20) | CT (n=20) | P-value |
| --- | --- | --- | --- |
| CIMT in left and right CCA | 0.63 (0.60-0.73) | 0.67 (0.56-0.79) | 0.76 |
| Maximum CIMT left and right CCA | 0.74 (0.71-0.90) | 0.83 (0.68-1.00) | 0.55 |
| Maximum CIMT left and right ICA | 1.11 (0.95-1.38) | 1.21 (0.90-1.42) | 0.89 |
| Difference between average and maximum CIMT in the left and right CCA | 0.14 (0.10-0.16) | 0.15 (0.13-0.18) | 0.16 |
| **After exclusion of current smokers** | CC (n=19) | CT (n=15) |  |
| CIMT in left and right CCA | 0.63 (0.60-0.74) | 0.67 (0.56-0.81) | 0.99 |
| Maximum CIMT left and right CCA | 0.75 (0.71-0.90) | 0.79 (0.68-1.03) | 0.78 |
| Maximum CIMT left and right ICA | 1.17 (0.96-1.40) | 1.16 (0.88-1.26) | 0.46 |
| Difference between average and maximum CIMT in the left and right CCA | 0.14 (0.10-0.17) | 0.15 (0.13-0.19) | 0.24 |
| **After exclusion of subjects treated with an anti-hypertensive drug** | CC (n=19) | CT (n=18) |  |
| CIMT in left and right CCA | 0.62 (0.60-0.74) | 0.65 (0.56-0.84) | 0.91 |
| Maximum CIMT left and right CCA | 0.74 (0.71-0.90) | 0.79 (0.68-1.04) | 0.60 |
| Maximum CIMT left and right ICA | 1.11 (0.94-1.34) | 1.16 (0.88-1.30) | 0.98 |
| Difference between average and maximum CIMT in the left and right CCA | 0.14 (0.10-0.16) | 0.15 (0.13-0.19) | 0.10 |
